# Targeted Training for Subspecialist Care in Children With Medical Complexity

**DOI:** 10.3389/fped.2022.851033

**Published:** 2022-05-16

**Authors:** Fabian Eibensteiner, Valentin Ritschl, Isabella Valent, Rebecca Michaela Schaup, Axana Hellmann, Lukas Kaltenegger, Lisa Daniel-Fischer, Krystell Oviedo Flores, Stefan Brandstaetter, Tanja Stamm, Eva Schaden, Christoph Aufricht, Michael Boehm

**Affiliations:** ^1^Division of Pediatric Nephrology and Gastroenterology, Department of Pediatrics and Adolescent Medicine, Comprehensive Center for Pediatrics, Medical University of Vienna, Vienna, Austria; ^2^Ludwig Boltzmann Institute Digital Health and Patient Safety, Medical University of Vienna, Vienna, Austria; ^3^Section for Outcomes Research, Center for Medical Statistics, Informatics and Intelligent Systems, Medical University of Vienna, Vienna, Austria; ^4^Ludwig Boltzmann Institute for Arthritis and Rehabilitation, Vienna, Austria; ^5^Center for Medical Statistics, Informatics and Intelligent Systems, Medical University of Vienna, Vienna, Austria; ^6^Christian Doppler Laboratory for Molecular Stress Research in Peritoneal Dialysis, Department of Pediatrics and Adolescent Medicine, Medical University of Vienna, Vienna, Austria; ^7^Division of Nephrology and Dialysis, Department of Medicine III, Medical University of Vienna, Vienna, Austria; ^8^Division of General Anaesthesia and Intensive Care Medicine, Department of Anaesthesia, Intensive Care Medicine and Pain Medicine, Medical University of Vienna, Vienna, Austria

**Keywords:** nephrology, medical education, training, medical complexity, children with medical complexity (CMC), residency, patient safety, children with chronic disease

## Abstract

**Background:**

Children with medical complexity (CMC) are prone to medical errors and longer hospital stays, while residents do not feel prepared to provide adequate medical care for this vulnerable population. No educational guidance for the training of future pediatric tertiary care specialists outside their field of expertise involving the multidisciplinary care of CMC exists. We investigated pediatric residents past educational needs and challenges to identify key learning content for future training involving care for CMC.

**Methods:**

This was a prospective mixed-methods study at a single pediatric tertiary care center. Qualitative semi-structured interviews with residents were conducted, submitted to thematic content analysis, linked to the American Board of Pediatrics (ABP) general pediatrics content outline, and analyzed with importance performance analysis (IPA). Quantitative validation was focused on key themes of pediatric nephrology within the scope of an online survey among pediatric residents and specialists.

**Results:**

A total of 16 interviews, median duration 69 min [interquartile range IQR 35], were conducted. The 280 listed themes of the ABP general pediatrics content outline were reduced to 165 themes, with 86% (theoretical) knowledge, 12% practical skills, and 2% soft skills. IPA identified 23 knowledge themes to be of high importance where improvement is necessary and deemed fruitful. Quantitative validation among 84 residents and specialists (response rate 55%) of key themes in nephrology yielded high agreement among specialists in pediatric nephrology but low interrater agreement among trainees and “trained” non-nephrologists. The occurrence of themes in the qualitative interviews and their calculated importance in the quantitative survey were highly correlated (tau = 0.57, *p* = 0.001). Two clusters of high importance for other pediatric specialties emerged together with a contextual cluster of frequent encounters in both in- and outpatient care.

**Conclusion:**

Regarding patient safety, this study revealed the heterogeneous aspects and the importance of training future pediatric tertiary care specialists outside their field of expertise involving the multidisciplinary care of CMC. Our results may lay the groundwork for future detailed analysis and development of training boot camps that might be able to aid the improvement of patient safety by decreasing preventable harm by medical errors, especially for vulnerable patient groups, such as CMC in tertiary care pediatrics.

## Introduction

Preventable harm in healthcare occurs in about 6% of patients across medical care settings (primary care, hospitals, intensive care units), with 12% leading to permanent disability or death (1). Medication and procedure-related errors, as well as nosocomial infections, are the most common adverse events in hospitals (2). Peaks of healthcare-related adverse events have been observed to reoccur annually during July and August in the United States and the United Kingdom, decreasing progressively after 1 month (3, 4). This tendency coincides with the enrolment of many new trainees and fellows at teaching hospitals, annually over 32,000 and 100,000 in Europe and the United States (5, 6). Such data suggest that current medical education does not sufficiently prepare graduates for their future work environment (3, 7–9).

Children with medical complexity (CMC) account for an increasing proportion of hospitalized pediatric patients resulting in about one-third of total pediatric healthcare costs (10–13). These patients are at particular risk for medical errors and longer hospital stays (14–18). Several studies suggest that residents often feel overwhelmed, anxious, and in doubt of their skills when caring for CMC (11, 19, 20). There is a high need for novel measures to equip pediatric residents with the knowledge and skill set to improve their performance in early clinical training, thus increasing patient safety (12).

Simulation-based boot camps at the beginning of clinical training might be an effective way to prepare young physicians for their future clinical environment (9, 21). A web-based multimedia curriculum for pediatric residents increased satisfaction, knowledge, behavior change related to verbal handoffs, and comfort with clinical care of CMC (18). This curriculum primarily focuses on the care of CMC with neurological impairment and technology dependence (e.g., spasticity, tracheostomy tubes).

In the care for CMC, important differences exist between Europe and the United States. In Europe, tertiary care center subspecialists care for all patients, including CMC. In contrast, in the United States, hospitalists mainly provide the care for these patients. Hospitalists are defined as “a physician whose primary professional focus is the general medical care of hospitalized patients and whose activities include patient care, teaching, research, and leadership related to hospital medicine” (22). Consequently, different needs and challenges for new residents becoming future pediatric subspecialists might prevail.

In the literature, several national educational curricula, syllabi, and content outlines for general pediatrics and pediatric subspecialties exist (23–28), in addition to individual efforts of needs assessments in particular areas of medicine for future general pediatricians, e.g., pediatric palliative care, pediatric gastroenterology curricula, or essential hypertension for primary care pediatricians (29–31). However, as Abbott and First concluded, a “one-size-fits-all” approach to pediatric residency training cannot be justified as training needs for subspecialty pediatricians and hospitalists primarily working in hospital-based medicine are sufficiently distinct from those of subspecialty pediatricians and general pediatricians working mainly in ambulatory medicine (32).

Currently, no national or international educational guidance for the training of future pediatric tertiary care specialists outside their field of expertise involving the multidisciplinary care of CMC exists. Some examples of similar schools of thought have been published in internal medicine (33–36), but to the best of our knowledge, no needs assessment for the training of residents becoming future subspecialists at a pediatric tertiary care center caring for CMC has been published yet. The primary objective of this mixed-methods study was to evaluate the pediatric resident's past educational needs and challenges during their clinical rotation in specific subspecialty areas of pediatrics (e.g., pediatric nephrology, pediatric cardiology) and to define key learning content for future resident and subspecialty training involving care for CMC.

## Methods

### Setting

The Comprehensive Center for Pediatrics at the Medical University of Vienna is the largest Austrian academic tertiary care center that focuses almost exclusively on caring for children with severe or orphan acute and chronic diseases in both inpatient and outpatient medicine. The Austrian pediatric residency curriculum is set for 6 years. At our center, all residents need to accomplish their training at the same, a total of six, different specialized divisions (“rotations;” pulmonology, allergology, and endocrinology; neurooncology and epileptology; cardiology and hemostaseology; nephrology, gastroenterology, and rheumatology; pediatric intensive care medicine and neonatology; pediatric ambulatory care) to learn the whole spectrum of pediatrics, with approximately 80% inpatient and 20% outpatient care.

A prospective mixed-methods study was conducted at the Department of Pediatrics and Adolescent Medicine. This study consisted of two phases, namely, (1) qualitative explorative interviews with a convenience sample of pediatric residents at different stages of clinical training to explore and evaluate educational needs and challenges yielding a defined set of competencies in the form of knowledge, practical skills, and soft skills in specific highly specialized work environments; and (2) quantitative validation (online survey) with all pediatric residents and specialists to rank these individual competencies through a larger-scale survey to create a more profound content outline. Approval was granted by our university's data protection agency beforehand.

### Qualitative Explorative Interviews

Qualitative explorative interviews were conducted from November 2019 until February 2020 by two researchers (FE and AH), supervised by an expert in qualitative research (VR). At the time of screening (10/2019), 58 residents were potentially eligible for inclusion in phase I of this study. In detail, we conducted open-ended, semi-structured individual interviews to allow in-depth discussion in a private setting to talk about their perceived resources and demands (37). Our developed and piloted semi-structured interview guide is given in the Supplementary Material. The interviews were audio-recorded and transcribed verbatim. Analysis of the collected data was conducted utilizing a qualitative thematic content analysis to identify codes (37, 38). First, an initial code list was created by reading through the collected data. Codes are defined as words that act as labels for specific concepts and describe the meaning of a piece of text (38). For each coding unit, two different labels were assigned, namely, theme and group. Theme refers to the content of the coded segment, and group to the type of learning by context (“knowledge,” “practical skill,” “soft skill”) and category refers to whether the theme of the coded unit was or was not learned by the resident during their rotation (“learned” vs. “missing,” respectively), as seen in the context of the coded unit. Examples are given in Table 1.

**Table 1:** Coding examples of the qualitative thematic content analysis.

**Quote**	**Codes**
“*[…] on ward number seven, blood gas analysis was a major topic, as our attendings actively made sure, that we were learning this topic. Furthermore, during this time, nephrologists held lectures on this topic for us […]”*	Theme = blood gas analysis Group = knowledge Category = learned
“*[…] I would have hoped to get some critical care training early on, especially on how to sedate a child […]”*	Theme = procedural sedation Group = practical skill Category = missing
“*[…] as a chronically ill and multimorbid child was dying, which came to our ward specifically for palliative care, I did not know what to do […]”*	Theme = end of life Group = soft skill Category = missing
“*[…] and hemolytic uremic syndrome (HUS) is also a one of the classics, which is frequently seen on our nephrology ward, because HUS occurs every few months […]”*	Theme = hemolytic uremic syndrome Group = knowledge Category = learned

To introduce better reproducibility and international standardization of our results, the original coded themes were linked to corresponding domains and subdomains as outlined in the American Board of Pediatrics (ABP) content outline created for the Pediatrics Board Examination in the United States. These content outlines drafted by content experts are regularly updated and available for different subspecialties, e.g., general pediatrics and pediatric nephrology (25–27). The content outlines are structured in a major content domain and several subdomains, e.g., content domain—*glomerular disorders*; subdomain—*nephropathies*; lower-order subdomain—*minimal change disease and variants* (27). We linked our previously identified themes to these content domains and subdomains. In a few cases with added descriptors, the lowest appropriate content domain or subdomain was chosen to preserve the meaning of the themes in context with the interview's original quotes. This is a modified approach of the previously published rules for International Classification of Functioning, Disability and Health linking in qualitative research (39). Themes that fit into more than one subspecialty and themes where apparent linking was not possible were discussed between two researchers and a pediatrician and then consistently categorized. For example, the theme *hemolytic uremic syndrome (HUS)* (as given in Table 1) was linked to the lowest appropriate subdomain *thrombotic microangiopathies* within the ABP content outline, categorized within *pediatric nephrology* > *glomerular disorders* > *nephropathies with systemic disease* (27). To ensure validity of our qualitative data collection and analysis, the following strategies were embodied from the beginning until the completion of our study, namely, (1) audit trail to ensure optimal replicability (40), (2) peer review/debriefing with specialists from our department's education committee (41, 42), and (3) reflexivity of executive researchers (40, 43).

**Figure 1:**
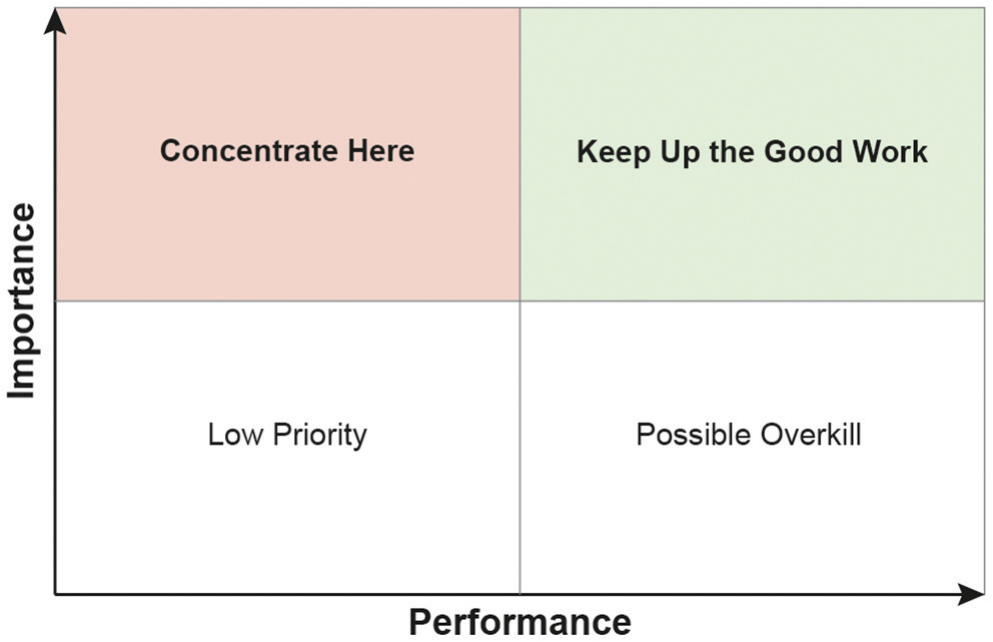
Importance performance analysis (IPA = needs assessment) adapted from Park et al. (44). The importance of each theme is derived from the frequency (number of occurrences = in how many interviews it was coded), the performance by the categorization (learned vs. missing).

After initial analysis, we displayed the emerged themes according to Park's et al. model of the importance performance analysis (IPA, = needs assessment) (44), which aids to identify key themes of high importance with low performance, where focused improvement is needed and deemed to be fruitful (see Figure 1).

### Quantitative Validation

To test the validity of this qualitative IPA, we set out to identify key themes within the IPA's results for focused interventions by a quantitative assessment. As pediatric nephrology was one of the three topics with the most identified themes of our IPA, we concentrated further investigations on this subspecialty: We submitted the 20 most frequently mentioned themes in pediatric nephrology to an online ranking among all physicians (= residents and specialists), utilizing SoSci Survey (SoSci Survey GmbH, Munich, Germany) (45). Each participant was asked to select the five top relevant (“TOP 5”) and the five least relevant (“LEAST 5”) themes (with the remaining 10 themes categorized as “Intermediate”). Participants were explicitly asked to assign the relative importance of each theme for training to become future tertiary care hospitalists outside pediatric nephrology. Furthermore, participants were asked to state their level of training (resident vs. specialist), and specialists were asked to declare their field of expertise. Survey invitations were emitted on March 1, 2021, and were online for 4 weeks. Participants received two reminders (after 1 and 3 weeks) to increase attendance.

### Statistical Analysis

Descriptive data were analyzed using absolute and relative frequencies, median and interquartile range (IQR), or mean and standard deviation (SD) depending on data distribution. For each theme submitted to the survey, an importance score (IS) was calculated as the mean reciprocal rank, defined as the mean of 1 over the ranking assigned by each participant, in accordance with similar qualitative research in pediatric nephrology (46). For example, if a theme was ranked into the “TOP 5” category by one participant and in “LEAST 5” by another participant, the IS for this theme would be calculated as the mean of 1/1 and 1/3, resulting in an IS of 0.67, respectively. Differences of IS between groups were calculated by utilizing Student's *t*-test as IS represent mean reciprocal ranks. Kendall's *W* was calculated to assess interrater agreement among participants. Kendall's rank correlation was used to calculate correlation coefficients. All themes were submitted to cluster analysis by calculating Euclidean distances of IS across different groups of subspecialties and residents and submitting them to hierarchical Ward clustering. Subspecialties were analyzed when at least four specialists of the respective subspecialty participated in our survey or categorized in “other.” Numbers of final clusters were evaluated based on contextual meaningfulness and discussed with experts in pediatric nephrology. Prioritization of themes and interrater agreement was further analyzed concerning experience, pooled by different training levels [Trainees (= residents), “trained” (= specialists outside pediatric nephrology), trainers [=specialists in pediatric nephrology)]. Statistical analysis was performed using IBM SPSS version 24.0.0.0 2016 (SPSS, Inc., Chicago, IL, USA) and R software (R Core Team 2020) (47).

## Results

### Qualitative Explorative Interviews

Over 4 months, 16 interviews were conducted with 16 residents [10 women, median age 30 years (IQR4)]. Baseline characteristics of the total and the studied population were similar, with 38 and 31% of residents based on neonatology and pediatric intensive care units, and 62 and 69% based on general wards and intermediate care units, respectively. Furthermore, sex distribution among the targeted and studied population was comparable, with 63 and 64% female residents, respectively. With a median number of in-house ward rotations of 3 (IQR 3), our study population accurately represents an equally distributed spectrum of residents with a wide and heterogeneous clinical exposure on different stages of their education. This is being indicated by the range of interviewed residents from early training up to almost completion of residency with a median at “half-time” (= 3 rotations).

The median duration of individual interviews was 69 min (IQR 35 min). Participants mentioned a mean of 28 (SD ± 13) themes in their interviews. In total, 165 unique themes were identified, resulting in a mean of 10 (SD ± 7) unique themes per participant. In addition, 86% (141/165) of themes were assigned to (theoretical) knowledge, 12% (20/165) to practical skills, and 2% (4/165) to soft skills. Of these themes, 62% (122/197) were assigned as sufficiently trained (category: learned) and 38% (75/197) to need additional training (category: missing), with double mentions by different participants. Assignment to these groups did not differ between ages, numbers of in-house rotations, and sex. Categorization of emerged themes into various pediatric subspecialties is displayed in Supplementary Table 1.

Practical skills mentioned by at least four interviewees (25%) are displayed in Table 2 and congregate into three distinct categories, namely, *resuscitation, sonography*, and *vascular/cerebrospinal fluid access*. Practical skills mentioned by <4 interviewees were *bladder catheterization* (19%, 3/16), *blood products transfusions* (19%, 3/16), *clinical presentation in neurology* (19%, 3/16), *incision and drainage* (19%, 3/16), and *transport management in critical care medicine* (13%, 2/16). Soft skills mentioned by at least two interviewees were *patient-parent-pediatrician relationship* (56%, 9/16) and *end-of-life care* (13%, 2/16).

**Table 2:** Practical skills (mentioned by at least four interviewees) drafted from the qualitative explorative interviews (*n* = 16).

**Practical skills**	***N* (%)**
**Resuscitation**	
Resuscitation	10 (63%)
Airway management	6 (38%)
Procedural sedation	5 (31%)
Stabilization and transition of newborn infants	5 (31%)
**Sonography**	
Point of care ultrasound (abdomen/emergency)	6 (38%)
Neuroimaging studies (sonography)	5 (31%)
Echocardiography (basic)	5 (31%)
**Vascular/cerebrospinal fluid access**	
Peripheral intravenous placement	16 (100%)
Central venous catheterization and handling	5 (31%)
Lumbar puncture	4 (25%)

The IPA of all emerged themes of our qualitative explorative interviews is displayed in Supplementary Figure 2. Table 3 shows the extracted 23 (16%) key knowledge themes from 10 different pediatric subspecialties in the high-importance and low-performance panel, i.e., focused improvement is needed and deemed fruitful. In total, 20 themes of pediatric nephrology emerged during the qualitative explorative interviews (see Supplementary Table 2). In addition, 85% (17/20) of these themes were correctly identified by the IPA. Three themes (*urinary tract infection, bacteriuria, and pyuria*; *acid-base disorders; hematuria and proteinuria*) were correctly identified within the high-importance and low-performance panel, as occurrence of missing was higher than learned. Two themes (*core diagnostics in nephrology; chronic kidney disease and end-stage kidney disease*) that were 100% missing were not highlighted by the IPA as they were mentioned once only. The IPA also highlighted the theme of *sodium and water balance* although the majority declared it as learned (missing:learned = 3:6).

**Table 3:** Extracted key knowledge themes from the importance performance analysis (IPA) high-importance and low-performance panel (*n* = 23) ranked by their importance (= number of occurrence) with nephrology themes in bold.

**Importance (ranked by**	**Theme**	**Assigned**
**number of occurrence)**		**subspecialty**
5	General pediatrics	General pediatrics
5	Inborn errors of metabolism	General pediatrics
4	Intrathoracic respiratory infections	Pulmonology
**3**	**Sodium and water balance**	**Nephrology**
3	Normal growth and development	General pediatrics
3	Clinical dermatologic presentation	General pediatrics
3	Age-Appropriate medical screenings	General pediatrics
3	Handoffs across the continuum of care	Hospital medicine
3	Emergency conditions	Emergency medicine
**3**	**Hematuria and proteinuria**	**Nephrology**
2	Diarrhea	Gastroenterology
2	Seizures	Emergency medicine
2	Principles of chemotherapy	Hematology-oncology
2	Supraventricular arrhythmias	Cardiology
**2**	**Acid-base disorders**	**Nephrology**
2	Disorders of endocrinology	Endocrinology
2	Antimicrobial stewardship principles	Infectious diseases
2	Asthma	Pulmonology
2	Insulin deficiency with hyperglycemia	Endocrinology
2	Chronic diarrhea	Gastroenterology
2	Failure to thrive	Hospital medicine
**2**	**Urinary tract infection, bacteriuria, and pyuria**	**Nephrology**
2	Gastrointestinal bleeding	Gastroenterology

### Quantitative Validation

Survey invitations were sent to all residents and specialists (*n* = 154). The response rate was 55% (84/154), with 14 being excluded due to doubled or incomplete entries, or due to ranking themes into “TOP 5” and “LEAST 5” categories simultaneously. A total of 70 responses (46%) could be included in our final analysis.

Of these, 46% (32/70) were residents and 54% (38/70) specialists, reflecting a mild overrepresentation of residents of the targeted medical staff (38% residents, 62% specialists). The remaining subspecialties were pooled and analyzed as a single category, namely, “other.” The distribution of specialists among subspecialties within the targeted (*n* = 84) and the surveyed (*n* = 38) population were similar, with 32 and 34% from neonatology and pediatric critical care, 18 and 11% from pediatric cardiology, 10 and 11% from pediatric nephrology, 7 and 11% from pediatric pulmonology, and 33 and 34% from “other.”

**Figure 2:**
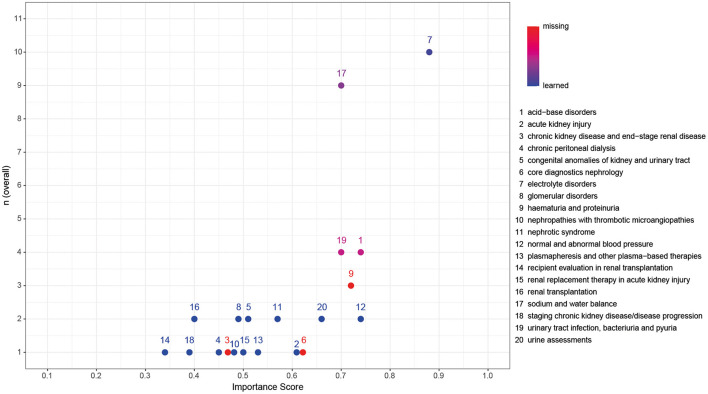
Validation of the importance of key nephrology themes within the qualitative interviews with the calculated importance scores (IS) of the quantitative survey. Color coding represents classification as the ratio of missing:learned, with red representing 100% missing and blue representing 100% learned. Kendall's rank correlation tau = 0.57, *p* = 0.001.

Figure 2 represents a significant high correlation between the occurrence of the theme in the qualitative interviews and the calculated IS with themes being assorted based on importance in the quantitative survey (tau = 0.57, *p* = 0.001). Themes of high importance in the qualitative interviews also display high importance within the quantitative survey. In contrast, themes that may be underrepresented in the interviews due to the lower number of participants show greater diversity along with the calculated ISs. Most importantly, *core diagnostics nephrology* and *chronic kidney disease and end-stage kidney disease* that the IPA, although classified as 100% “missing,” failed to identify as important were assigned with higher IS within the quantitative validation.

Overall interrater agreement on IS was poor (*W* = 0.3, *p* < 0.001), as well as between residents (*W* = 0.32, *p* < 0.001) and “trained” (*W* = 0.3, *p* < 0.001). The highest interrater agreement was achieved between trainers (*W* = 0.66, *p* < 0.001).

**Figure 3:**
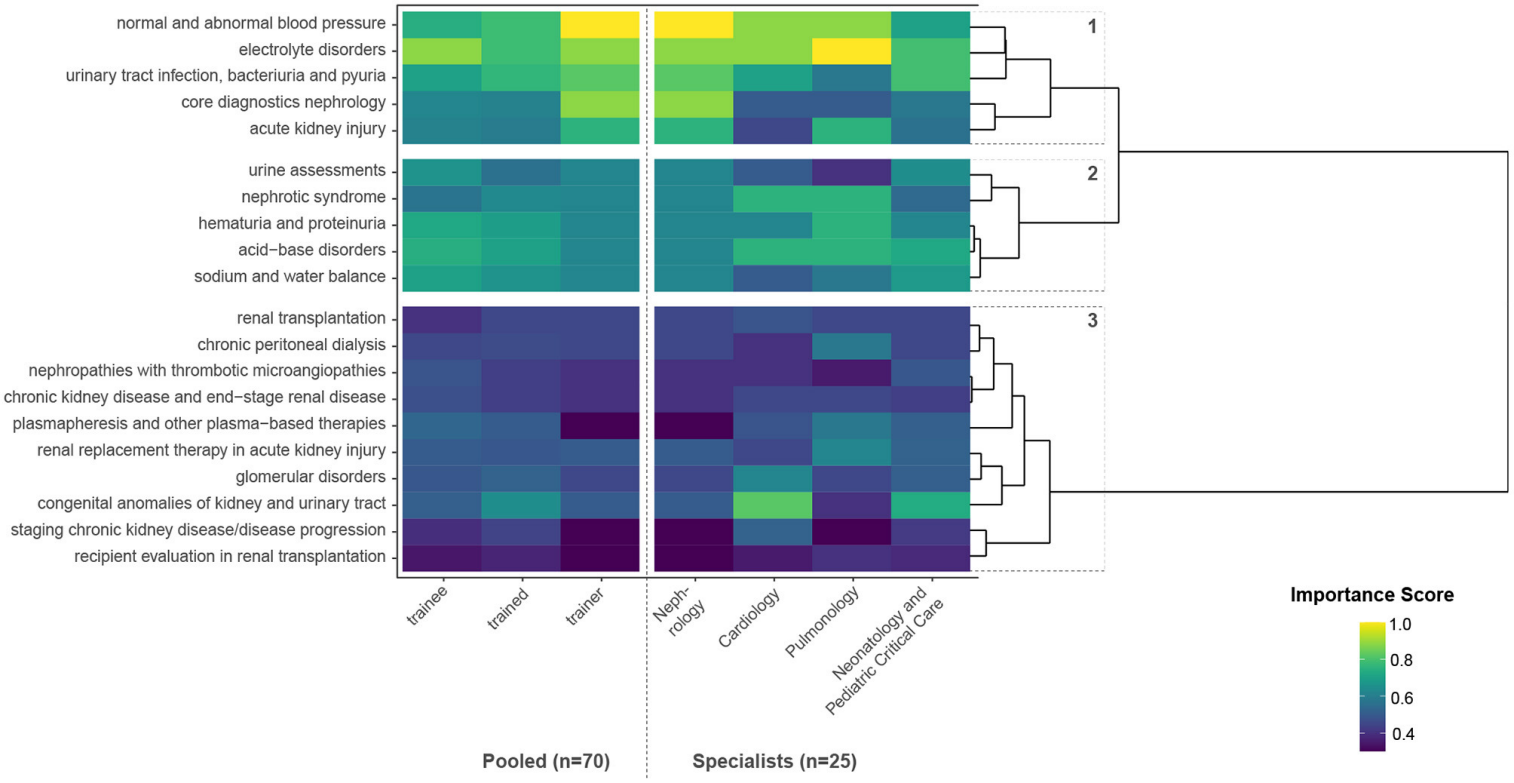
Hierarchical clustering of the most frequently mentioned themes in pediatric nephrology by importance scores (IS) of trainees, “trained,” and trainers, as well as different specialists.

Hierarchical clustering of the most frequently mentioned themes in pediatric nephrology by IS of trainees, “trained,” and trainers resulted in three major contextual clusters, as displayed in Figure 3. Cluster number 1 representing the most important themes for future tertiary care hospitalists is *normal and abnormal blood pressure, electrolyte disorders, urinary tract infection, bacteriuria, and pyuria, core diagnostics nephrology*, and *acute kidney injury*. Cluster number 2 represents a total of five further themes of high importance, resulting in a contextual cluster of frequent encounters in both inpatient and outpatient care (*urine assessments, nephrotic syndrome, hematuria and proteinuria, acid-base disorders*, and *sodium and water balance*). Together, clusters 1 and 2 cover both the themes identified by the IPA and one of the two themes not identified by the IPA, although classified as 100% “missing” (*core diagnostics nephrology*).

Although classified as 100% “missing” within the qualitative interviews, *chronic kidney disease and end-stage kidney disease* was not rated with high importance within the quantitative survey, neither by trainees, “trained,” nor by trainers. Cluster number 3 represents themes where in-depth knowledge and specialization is needed and consists of themes related to end-stage kidney disease, renal replacement therapy, other extracorporeal treatments, and complex kidney diseases. In subanalysis, the theme *CAKUT (congenital anomalies of the kidney and urinary tract)* is rated with high importance by pediatric cardiologists and neonatologists/intensivists.

Representative quotes of the themes identified in both most important clusters (1 and 2) describing clinical situations where the importance of these themes was stated are given in Supplementary Table 3.

Supplementary Figure 3 represents the calculated IS for different training levels [Trainees (= residents), “trained” (= specialists outside pediatric nephrology), trainers (= specialists in pediatric nephrology)] themes being assorted on the basis of importance, with decreasing importance from left to right. Statistical comparison of IS is displayed in Table 3.

## Discussion

The aim of this explorative mixed-methods study was to evaluate educational needs and challenges faced by pediatric residents yielding competencies of knowledge, and practical and soft skills at an academic pediatric tertiary care center that focuses almost exclusively on caring for children with severe or orphan diseases. Several national curricula, outlines, and frameworks exist, defining the knowledge and skill set pediatricians should have acquired by the end of their residency or specialization training. However, the current literature does not offer consensus on specific training recommendations for interdisciplinary inpatient care for CMC with multiple-organ involvement (7, 14, 17, 18). Most importantly, a “one-size-fits-all” approach to pediatric residency training may not be appropriate as hospital-based pediatrics and pediatric primary care and ambulatory medicine are sufficiently different (32).

Our standardized qualitative analysis revealed the need for systematic incorporation of subject-specific clinical training in adjacent pediatric specialties for future highly specialized pediatric hospitalists caring for CMC with multiorgan disease. We reduced the 280 listed themes (= lowest domains) in the ABP content outline to 165 unique themes and finally extracted 23 key themes where improvement is necessary and deemed fruitful. A subset of key themes in pediatric nephrology was validated by importance for training of future hospitalists in pediatric tertiary care. Hierarchical clustering of nephrology themes resulted in two clusters of high importance dominated by *normal and abnormal blood pressure, electrolyte disorders, urinary tract infection, bacteriuria, and pyuria, core diagnostics nephrology*, and *acute kidney injury*, together with a contextual cluster of frequent encounters in both inpatient and outpatient care (*urine assessments, nephrotic syndrome, hematuria and proteinuria, acid-base disorders*, and *sodium and water balance*). The third cluster, including e.g., renal replacement therapy, was rated as of low importance, most probably because these patients are usually consulted and very closely monitored by pediatric nephrologists.

In this study, we present the results and a methodological framework based on the educational needs in pediatric nephrology for tertiary care hospitalists in training as key learning content for the development of new curricula and/or further studies on this matter.

### Heterogeneity of Medical Training Programs in Pediatrics

National educational content outlines are highly diverse and generally broad or specifically tailored to specialists needs. The Austrian training content outline for general pediatrics (48) consists of an obligatory basic training module and seven different additional modules (e.g., nephrology and urology; neuropediatrics, sleep medicine, psychosomatics), of which three must be chosen for in-depth clinical training. In contrast, the Royal College of Pediatrics and Child Health (RCPCH) general pediatrics syllabus for general pediatrics training in the United Kingdom (24) describes five general learning outcomes (e.g., resuscitation, team leading, and management) and a sixth learning outcome consisting of pediatric conditions from 14 areas of medicine. The US-focused ABP general pediatrics content outline (26) provides a more detailed description with 280 learning themes structured within 25 different content domains relating to several subspecialties of pediatric medicine.

### Practical and Soft Skills

The identified practical and soft skill set (Table 2) generally matches the Austrian training content outline well, whereas the ABP and RCPCH only mention few practical and soft skills. Details are given in Supplementary Table 4. Despite being required, *quality improvement/assurance* did not occur in our analysis, which is in line with recent literature where only one-third of pediatric residents reported positive educational experiences in quality improvement (49). The importance of mastering skills such as *resuscitation* (63%) and *stabilization and transition of newborn infants* (31%) is reflected by a pediatric tertiary care survey where 72–96% of third-year residents attended events of cardiopulmonary resuscitation during their clinical practice (50). Another important skill set being mentioned by 19% of participants is *blood products transfusions*. As a recent international evaluation among pediatric residents concluded, there is an urgent need for improved education in transfusion medicine as this large international surveyed group of pediatric residents performed poorly on a transfusion medicine knowledge exam (51).

In our analysis, *patient-parent-pediatrician relationship* was considered a relevant soft skill to be mastered by medical trainees, which is also included in the Accreditation Council for Graduate Medical Education (ACGME) milestones to trace pediatric residents' development but does not specifically appear in the RCPCH or ABP content outline. Notably, *end-of-life care*, despite being mentioned only in 13% of our interviews, yielded strong emotional responses by the interviewed residents, particularly anxiety as no uniform training had been established: “*[…] Our daily hand-off for this patient was, that he was terminal and could die anytime soon. The first thing that came to my mind was, I hope this does not happen during my shift. At 5 a.m., 21 h into my shift, my only thought was: 3 h left until hand-off, thank God. My own mindset, that fact that I thought this way, is awful to me. Ideally, I should be able to say: okay, I am well-trained with this kind of situation, and I am able to care and provide support to the patient and his/her family. […]”*. With more than 50% of deaths in childhood in the United States occurring in the hospital and only 39% of pediatric residents receiving adequate palliative care training (52), and as careful breakdown of our explorative qualitative analysis suggests, training in *end-of-life care* for pediatricians should be greatly enhanced. This has also been recommended by the American Academy of Pediatrics, the Institute of Medicine and the ACGME (52, 53). In line with other needs assessments specifically targeting pediatric palliative care, there is a clear need for increased efforts in pediatric palliative care education during residency training as residents perceived their training in palliative care to be inadequate with no improvement over time (30). Training future (pediatric) hospitalists in all of these skills is of great importance as some of these skills, e.g., blood products transfusions, are generally needed often and rarely produce adverse events, whereas others, e.g., *end-of-life care*, are needed rarely but have a high potential to go wrong.

### Importance Performance Analysis: Knowledge

The 23 knowledge themes identified by our IPA with high importance and potential for improvement are generally well-represented in all three content outlines and might thus be regarded as extracted key themes for targeted training. Details of consistency and differences between content outlines and qualitative interviews are given in Supplementary Table 5. Noteworthy, the Austrian content outline includes additional aspects important for the care of CMC, e.g., *developmental and social pediatrics, psychosomatics, ergotherapy, analgesia, palliative care, follow-up care*, and *transition to adult care*, that were not identified by our IPA. In contrast, *antimicrobial stewardship principles* and *handoffs across the continuum of care* were identified with high importance and low performance by our IPA but are not mentioned in the analyzed national educational content outlines, although the ACGME milestones include handoffs as an important skill for residents. Especially *antimicrobial stewardship principles* is of valuable interest to hospitalists caring of CMC as the prevalence of multidrug-resistant bacterial infections in children has significantly increased during the last two decades (54). CMC may receive many antibiotic treatments during their lifetime, and studies have shown that antimicrobial stewardship programs can be effectively applied in pediatric inpatient settings, e.g., neonatal care (55). Medical errors are estimated to be attributed in approximately 80% to communication breakdowns. In particular for CMC, standardized high-quality *handoffs across the continuum of care* are essential for patient safety. However, as the committee on hospital care of the AAP states, “Hand-off communication is a skill requiring training and practice. Attending physicians are likely to benefit from ongoing training and monitoring of a standard approach to hand-offs” (56). This verifies our findings and highlights the importance of placing great emphasis on teaching *antimicrobial stewardship principles* and *handoffs across the continuum of care* to future hospitalists in pediatric tertiary care.

Themes in pediatric nephrology labeled with high importance (clusters 1 and 2 of Figure 3) in our analysis are generally well-covered by the RCPCH and ABP content outlines. In the Austrian general pediatrics content outline, no consistent standard exists on specific nephrology training for general pediatricians, let alone hospitalists/specialists outside pediatric nephrology. Therefore, to assume that future non-nephrology specialists, especially at tertiary care centers caring for CMC, should get by with the nephrological knowledge of general pediatricians is utopian fallacy. Our results indicate high importance of training in diagnostic tools essential to nephrology as the themes *core diagnostics in nephrology* and *urine assessments* emerged in a high-importance cluster in addition to *hematuria and proteinuria*, whereas the RCPCH syllabus and ABP content outline cast no additional focus on diagnostics. However, the ABP content outline explicitly features *genetic disorders, diseases, and conditions* in pediatric nephrology, which did not emerge in our analysis. Arguably there has been tremendous progress in the knowledge on genetic backgrounds of kidney disease, also dramatically influencing diagnostic progress and therapeutic pathways, e.g., for patients suffering from steroid-resistant nephrotic syndrome with genetic origins (57). This is of special importance since, on the one hand, communicative skills in the setting of *patient-parent-pediatrician relationships* emerged as highly relevant soft skill for young residents, but, on the other hand, literature reviews conclude that especially to young pediatric residents communication of complex medical information, especially genetic information, can be very challenging, and education of pediatric residents in these skills should be reinforced (58).

### Quantitative Validation

The quantitative survey-based validation of key themes in pediatric nephrology of a subset of 20 identified nephrology themes revealed significantly high correlation (tau = 0.57, *p* = 0.001, as displayed in Figure 2). Themes of low importance display greater diversity along the calculated IS and may be underrepresented in the interviews due to a lower number of participants and free exploration of themes. Both themes classified as 100% “missing” and failed to be identified by the IPA received higher IS within the quantitative validation. This might reflect the implicit knowledge on diagnostic tools summarized in *core diagnostics nephrology* as well as for the care of children with *chronic kidney disease and end-stage kidney disease*. Themes of undoubtedly high importance, also after validation, were *sodium and water balance, electrolyte disorders*, and *acid-base disorders*, being an integral part of inpatient care.

As displayed in Figure 3, Supplementary Figure 3, and Table 4, perception of importance of themes between trainees, “trained,” and trainers was similar but not identical, likely reflecting distinct training needs for different subspecialties. Only the theme *normal and abnormal blood pressure* was ranked higher by trainers than by trainees, whereas other significant themes (*plasmapheresis and other plasma-based therapies, staging chronic kidney disease/progression*) were ranked lower by trainers, which might stem from the trainers' point of view of consultant requests in these areas. This comes with no surprise as similar needs assessments between pediatric residents and attending staff for education in clinical pharmacology also resulted in similar but not identical learning needs (59). Accordingly, interrater agreement of relevancy was high between pediatric nephrologists (= trainers) but low among trainees and “trained” (= i.e., non-nephrology specialists). This corroborates different emphasis of nephrological themes in diverse subspecialties of pediatrics as, for example, the importance of *core diagnostics in nephrology* is perceived significantly different among specialists of neonatology and pediatric critical care vs. pediatric nephrologists. However, as displayed in Figure 4 homogeneous importance of major thematic clusters of nephrological themes for non-nephrologists could be identified despite minor differences between specialties.

**Table 4:** Comparison of importance scores (IS) between trainees, “trained,” and trainers.

Theme*a	**Trainees, IS ±SD**	**“Trained,” IS ±SD**	*p*-value*b	**Trainers, IS ±SD**	*p*-value*c
Electrolyte disorders	0.88 ± 0.22	0.78 ± 0.27	0.1	0.88 ± 0.25	0.95
**Normal and abnormal blood pressure**	0.74 ± 0.29	0.78 ± 0.28	0.61	**1.0 ±0**	**<0.001**
Urine assessments	0.66 ± 0.28	0.56 ± 0.21	0.14	0.62 ± 0.25	0.83
Nephrotic syndrome	0.57 ± 0.2	0.63 ± 0.22	0.2	0.62 ± 0.25	0.67
Congenital anomalies of kidney and urinary tract	0.51 ± 0.21	0.64 ± 0.28	0.05	0.5 ± 0	0.73
**Plasmapheresis and other plasma-based therapies**	0.53 ± 0.22	0.5 ± 0.27	0.61	**0.3 ±0**	**<0.001**
Renal replacement therapy in acute kidney injury	0.5 ± 0.21	0.49 ± 0.21	0.91	0.5 ± 0	0.08
**Staging chronic kidney disease/ progression**	0.39 ± 0.15	0.44 ± 0.2	0.28	**0.3 ±0**	**0.002**
**Recipient evaluation in kidney transplantation**	0.34 ± 0.8	0.37 ± 0.14	0.4	**0.3 ±0**	**0.006**

* *aAll themes with a p < 0.2 are shown, themes with a p < 0.05 are given in bold;*

* *bStudent's t-test between trainees and “trained”;*

* *cStudent's t-test between trainees and trainers; p-values are unadjusted*.

Although the RCPCH syllabus includes *acute nephritis, chronic kidney disease*, and *enuresis* in their content outline, both *acute nephritis* and *chronic kidney disease* did not receive high ISs by non-nephrologists. This might stem from major involvement of pediatric nephrologists with these patients in the training environment of our tertiary care center since they represent a preselection of the most severe and complex cases. *Enuresis* did never emerge during our analysis as it being a generally common typical outpatient problem (60). *CAKUT*, being by far the major cause of chronic kidney disease (CKD) in childhood (48% of CKD), emerged as a topic of high importance to future non-nephrologist tertiary care specialists, especially for pediatric cardiologists (IS 0.8). These findings are not surprising given the association of *CAKUT* with congenital heart disease, e.g., DiGeorge syndrome (61). Given the high importance of *CAKUT* in multidisciplinary care, it seems surprising that this disease spectrum did not gain additional attention in the RCPCH syllabus.

### Strengths and Limitations

To the best of our knowledge, this is the first study evaluating this special niche of residency training with “needs assessment” based on qualitative interview exploration, refined by quantitative importance validation. While several national and international educational frameworks and guidelines for pediatrics and pediatric subspecialties exist, they do not focus on the multidisciplinary treatment of CMC with multiorgan diseases involving collaboration among subspecialties. Several examples underlining the importance of interdisciplinary exchange in highly specialized areas of medicine can be drawn from literature in internal medicine, e.g., dialysis for non-nephrologists (33, 34, 36), CKD for non-nephrologists (35), or heart- and lung transplantation with associated renal complications (62). In addition, renal involvement in mitochondrial cytopathies (63) and cross-over areas such as pediatric onconephrology (64) might warrant relevant benefits for nephrological knowledge in other highly specialized areas of pediatrics. As this study was initiated by members of the pediatric nephrology team, we decided to focus on this subspecialty for validation, although themes from cardiology and general pediatrics were more frequently mentioned. Qualitative research explores the unexpected in specific populations by holistically seeking to understand the participants' perspectives on the phenomena of interest and might be more feasible than standard methods of quantitative research when evaluating processes and outcomes of medical education (38, 65). Due to the COVID-19 pandemic, we were compelled to abort qualitative interviews after 4 months. Regarding this shortcoming, some residents were deprived to mention important aspects, highly relevant to them, and rare themes might not have been emerged and identified by our analytic approach. Despite a limited number of qualitative interviews, interviewed residents are generally representative for target population in terms of baseline characteristics. Small participant numbers in each subcategory (i.e., nephrologists, cardiologists) limit accuracy of comparison for drawn conclusions on the basis of our survey. However, our analysis is strengthened by a survey with high interrater agreement between specialists of the thematic focus of this work.

## Conclusion

This applied a mixed-methods approach to build key learning content for training needs of future subspecialty hospitalists caring for CMC, highlighting aspects of nephrology validated by an orthogonal method. A total of 280 listed themes within the ABP content outline were condensed to 23 key themes of high importance in need for improvement by an IPA, as well as to key themes in pediatric nephrology validated in a larger cohort for importance for training of future pediatric tertiary care hospitalists. Most importantly, future training should emphasize important aspects of patient safety beyond subspecialty training, such as *antimicrobial stewardship principles* and *handoffs across the continuum of care*, practical skills, such as *point-of-care sonography* and *blood products transfusions*, as well as *end-of-life care*. This study also introduces hierarchical clustering analysis to further tailor educational contents for a given subspecialty to distinct needs of pediatricians in training for another subspecialty. The knowledge basis and used methodologies of this study may lay the groundwork for future detailed analysis and the development of digital boot camps and might be able to aid the improvement of patient safety by decreasing preventable harm by medical errors, especially for vulnerable patient groups, such as CMC in tertiary care pediatrics.

## Data Availability Statement

The original contributions presented in the study are included in the article/Supplementary Material, further inquiries can be directed to the corresponding author/s.

## Ethics Statement

Ethical review and approval was not required for the study on human participants in accordance with the local legislation and institutional requirements. The patients/participants provided their written informed consent to participate in this study.

## Author Contributions

FE, CA, and MB developed concept and design, had full access to all the data in the study, take responsibility for the integrity and accuracy of the data and subsequent analysis, drafted the initial manuscript, conducted analysis and interpretation of data, and reviewed and revised the manuscript. IV, RS, AH, VR, and SB conducted further data analysis and interpretation. FE, AH, VR, IV, and RS collected all data presented in this manuscript. LK, LD-F, TS, KO, and ES performed critical revision for the manuscript for important intellectual content. All authors approved the final manuscript as submitted.

## Funding

This research was funded by the Anniversary Fund of the Oesterreichische Nationalbank (OeNB) Nr. 17194.

## Conflict of Interest

The authors declare that the research was conducted in the absence of any commercial or financial relationships that could be construed as a potential conflict of interest.

## Publisher's Note

All claims expressed in this article are solely those of the authors and do not necessarily represent those of their affiliated organizations, or those of the publisher, the editors and the reviewers. Any product that may be evaluated in this article, or claim that may be made by its manufacturer, is not guaranteed or endorsed by the publisher.
