# Supplementary material for: Targeted Training for Subspecialist Care in Children With Medical Complexity
Source: Front Pediatr. 2022 May 16;10:851033. doi: 10.3389/fped.2022.851033 (PMC9149215; doi:10.3389/fped.2022.851033)
Supplement: Supplementary file 1 [file Table_1.DOCX]

**Supplemental Table 1. Themes (n=165) drafted from the qualitative explorative interviews categorized by pediatric subspecialty.**

| **Pediatric subspecialty** | **N (%)** |
| --- | --- |
| Cardiology | 29 (18%) |
| General Pediatrics | 22 (13%) |
| Nephrology | 20 (12%) |
| Emergency Medicine | 16 (10%) |
| Gastroenterology | 14 (9%) |
| Hospital Medicine | 13 (8%) |
| Critical Care Medicine | 11 (7%) |
| Pulmonology | 10 (6%) |
| Endocrinology | 6 (4%) |
| Neonatal-Perinatal Medicine | 6 (4%) |
| Hematology-Oncology | 5 (3%) |
| Developmental-Behavioral | 5 (3%) |
| Infectious Diseases | 3 (2%) |
| Neurology | 2 (1%) |
| Rheumatology | 2 (1%) |
| Medical Toxicology | 1 (1%) |
