# Supplementary material for: Targeted Training for Subspecialist Care in Children With Medical Complexity
Source: Front Pediatr. 2022 May 16;10:851033. doi: 10.3389/fped.2022.851033 (PMC9149215; doi:10.3389/fped.2022.851033)
Supplement: Supplementary file 2 [file Table_2.DOCX]

**Supplemental Table 2. Top 20 nephrological themes drafted from the qualitative explorative interviews (n=16) ranked by their importance (=number of occurrence). Correctly identified themes are marked in bold.**

| **Importance (ranked by number of occurrence)** | **Theme** | **Missing:learned** | **IPA high importance and low performance panel** |
| --- | --- | --- | --- |
| 10 | Electrolyte disorders | 1:9 | No |
| 9 | Sodium and water balance | 3:6 | Yes |
| **4** | **Urinary tract infection, bacteriuria, and pyuria** | **2:2** | **Yes** |
| **4** | **Acid-base disorders** | **2:2** | **Yes** |
| **3** | **Hematuria and proteinuria** | **3:0** | **Yes** |
| 2 | Urine assessments | 0:2 | No |
| 2 | Kidney transplantation | 0:2 | No |
| 2 | Normal and abnormal blood pressure | 0:2 | No |
| 2 | Nephrotic syndrome | 0:2 | No |
| 2 | Glomerular disorders | 0:2 | No |
| 2 | Congenital anomalies of kidney and urinary tract | 0:2 | No |
| 1 | Renal replacement therapy in acute kidney injury | 0:1 | No |
| 1 | Nephropathies with thrombotic microangiopathies | 0:1 | No |
| 1 | Staging chronic kidney disease/disease progression | 0:1 | No |
| 1 | Recipient evaluation in kidney transplantation | 0:1 | No |
| 1 | Plasmapheresis and other plasma-based therapies | 0:1 | No |
| **1** | **Core diagnostics in nephrology** | **1:0** | **No** |
| 1 | Chronic peritoneal dialysis | 0:1 | No |
| **1** | **Chronic kidney disease and end-stage kidney disease** | **1:0** | **No** |
| 1 | Acute kidney injury | 0:1 | No |

*IPA = importance performance analysis (=needs analysis).*
