# Supplementary material for: Targeted Training for Subspecialist Care in Children With Medical Complexity
Source: Front Pediatr. 2022 May 16;10:851033. doi: 10.3389/fped.2022.851033 (PMC9149215; doi:10.3389/fped.2022.851033)
Supplement: Supplementary file 3 [file Table_3.DOCX]

**Supplemental Table 3. Representative quotes of the themes identified in both most important clusters (one and two) describing clinical situations where importance of the themes was stated.**

| **Theme** | **Quote** |
| --- | --- |
| **Cluster one** |  |
| Normal and abnormal blood pressure | *[…] on this ward you learn everything about blood pressure. […]* |
| Electrolyte disorders | *[…] “prescribe this patient this electrolyte infusion” – you never did it before, but you sit there, and you calculate. […] I really learned how to deal with electrolytes on this ward. […]* |
| Urinary tract infection, bacteriuria, and pyuria | *[…] how to deal with a urinary tract infection of a patient with chronic kidney disease. […]* |
| Core diagnostics nephrology | *[…] Call the nephrologists - what should we do, what parameters do they want, do we need special parameters, because you cannot know everything. [...]* |
| Acute kidney injury | *[…] you then begin to understand, that this patient is now, additionally to his rare disease, suffering from in acute kidney injury and is placed on the cardiology ward due to his primary disease. […]* |
| **Cluster two** |  |
| Urine assessments | *[…] urine assessments are an important topic. […] After being on this ward I had the feeling that I understood urinary electrolytes and different types of proteinuria slightly more. […]* |
| Nephrotic syndrome | *[...] on this ward you certainly gain insight into nephrotic and /nephritic syndrome. […]* |
| Hematuria and proteinuria | *[...]and then there comes a patient with proteinuria. When do I have to worry, when do I need to initiate which kind of diagnostic? […]* |
| Acid-base disorders | *[…] on this ward blood gas analysis was often a hot topic. The attendings wanted us to understand and learn it – and sometimes there were also presentations by the nephrologists on this topic. […]* |
| Sodium and water balance | *[…] how to calculate and deal with a patient’s fluid balance – this is something I learned on the last presentation/training. […]* |
