# Supplementary material for: Targeted Training for Subspecialist Care in Children With Medical Complexity
Source: Front Pediatr. 2022 May 16;10:851033. doi: 10.3389/fped.2022.851033 (PMC9149215; doi:10.3389/fped.2022.851033)
Supplement: Supplementary file 4 [file Table_4.DOCX]

**Supplementary Table 4. Important consistency and differences between national educational content outlines and the results of our qualitative explorative interviews – practical skills.**

| **Theme** | **Austrian training content outline (48)** | **RCPCH syllabus (24)** | | **ABP content outline (26)** |
| --- | --- | --- | --- | --- |
| **Themes identified in the national educational content outlines and our qualitative interviews** | | | | |
| *Resuscitation* | X | X | | X |
| *Stabilization and transition of newborn infants* | X | X | | X |
| *Blood products transfusions* | X |  | |  |
| **Themes identified in our qualitative interviews but missing in the national educational content outlines** | | | | |
| *Lumbar puncture* | X | X | X | |
| *Neuroimaging studies (sonography)* | X | X | X | |
| *Echocardiography (basic)* | X | X | X | |
| *Point of care ultrasound (abdomen/emergency)* | X | X | X | |
| *Central venous catheterization and handling* | X | X | X | |
| **Themes identified in the national educational content outlines but did not, or only once, occur in our qualitative interviews** | | | | |
| *General pediatric care* (e.g., wound care, securing and documenting evidence for forensics, removal of nasal foreign bodies) | X | No other specific practical skills are mentioned. | | |
| *Quality improvement/assurance* | X |  |  |  |

*RCPCH = Royal College of Paediatrics and Child Health; ABP = American Board of Pediatrics.*
