# Supplementary material for: Targeted Training for Subspecialist Care in Children With Medical Complexity
Source: Front Pediatr. 2022 May 16;10:851033. doi: 10.3389/fped.2022.851033 (PMC9149215; doi:10.3389/fped.2022.851033)
Supplement: Supplementary file 5 [file Table_5.DOCX]

**Supplementary Table 5. Important consistency and differences between national educational content outlines and the results of our qualitative explorative interviews – knowledge.**

| **Theme** | **Austrian training content outline (48)** | **RCPCH syllabus (24)** | | **ABP content outline (26)** |
| --- | --- | --- | --- | --- |
| **Themes identified in our qualitative interviews but missing in the national educational content outlines** | | | | |
| *Antimicrobial stewardship principles* | X | X | | X |
| *Hand-offs across the continuum of care* | X | X | | X |
| *Normal growth and development* |  | X | |  |
| *Age-appropriate medical screenings* |  | X | |  |
| *Principles of chemotherapy* | X | X | | X |
| *Gastrointestinal bleeding* | X |  | | X |
| **Themes identified in the national educational content outlines but did not, or only once, occur in our qualitative interviews** | | | | |
| *Cross-sectional areas of (pediatric) medicine* (e.g., mental and behavioral health; orthopedics and sports medicine; eye, ear, nose and throat) |  | (X) | X | |

*RCPCH = Royal College of Paediatrics and Child Health; ABP = American Board of Pediatrics.*
