# Supplementary material for: Targeted Training for Subspecialist Care in Children With Medical Complexity
Source: Front Pediatr. 2022 May 16;10:851033. doi: 10.3389/fped.2022.851033 (PMC9149215; doi:10.3389/fped.2022.851033)
Supplement: Supplementary file 6 [file Image_1.PDF]

Supplemental Figure 1. Interview guide (translated version, original in German).

Questions in italic font where not subject of this study and will be analyzed and reported in subsequent studies.

1. When you think about your first day at the Children's Hospital, what comes to mind?
2. *What were your expectations of clinical training in the various areas of the Children's Hospital?*
    a. *Were these expectations met?*
3. What do you consider to be basic requirements that one should have for training in the different areas of the Children's Hospital?
4. What knowledge or skills were most helpful to you in your clinical training in the various areas of the Children's Hospital?
5. What knowledge or skills would have been more helpful at the beginning of your clinical training in the different areas of the Children's Hospital?
6. Which knowledge or skills gave you confidence to work independently in the daily clinical routine in the different areas of the Children's Hospital?
7. What particularly "challenging situations" did you experience in the clinical routine in the different areas of the Children's Hospital?
    a. In what situations have you felt unprepared or overwhelmed?
    b. Were there also challenging situations that you grew from?
8. In what situations did you feel that your knowledge or skills in the various clinical areas of the Children's Hospital were not yet adequate?
9. What were the common difficulties encountered in the daily clinical routine at the wards of the different clinical areas of the Children's Hospital?
    a. Concerning the patients
    b. *Concerning the team*
    c. *Concerning the cooperation with other clinical areas*
10. *How did you perceive your own role in the team of the wards of the different clinical areas of the Children's Hospital?*
    a. *Regarding the hierarchy*
    b. *Regarding integration into the team*

11. *What allowed you to learn the most about each of the specialty-specific topics in the different clinical areas of the Children's Hospital?*
    a. *Case discussions*
    b. *Textbooks*
    c. *Digital resources*
    d. *Patient care or rounds*
    e. *Working under the direction of the attending physician*
    f. *By working independently*
12. *What motivated you to acquire more knowledge about specialty-specific topics in the various clinical areas of the Children's Hospital?*
    a. *Special patients*
    b. *Special incidents*
    c. *Other colleagues*
13. *Do you feel that in the clinical setting, stress or lack of time has affected your training?*
14. What do you wish you had known/been able to do before you were first called to see patients on night duty?
15. What particularly "challenging situations" did you experience on night duty?
16. *Thinking back, do you feel that you had a supporting role in your clinical education?*
    a. *Regarding your own contribution to learning*
    b. *What did this own contribution look like?*
17. What teaching content/focus topics do you particularly remember being taught during your training in the various areas of the pediatric clinic?
18. With regard to the training of future pediatric specialists, where do you see positive potential in the various areas of the Children's Hospital that can be built upon?
    a. What still needs to be improved?
19. Are there any comments on your part that you would still like to mention, and which have not yet been discussed in the interview?
    a. Ideas regarding training?
    b. Visions for the future
    c. Other topics
