# Supplementary material for: Targeted Training for Subspecialist Care in Children With Medical Complexity
Source: Front Pediatr. 2022 May 16;10:851033. doi: 10.3389/fped.2022.851033 (PMC9149215; doi:10.3389/fped.2022.851033)

Supplemental Figure 3. Importance Score of themes rated by trainees (=residents, n=32), "trained" (=specialists outside pediatric nephrology, n=38), and trainers (=specialists in pediatric nephrology, n=4).

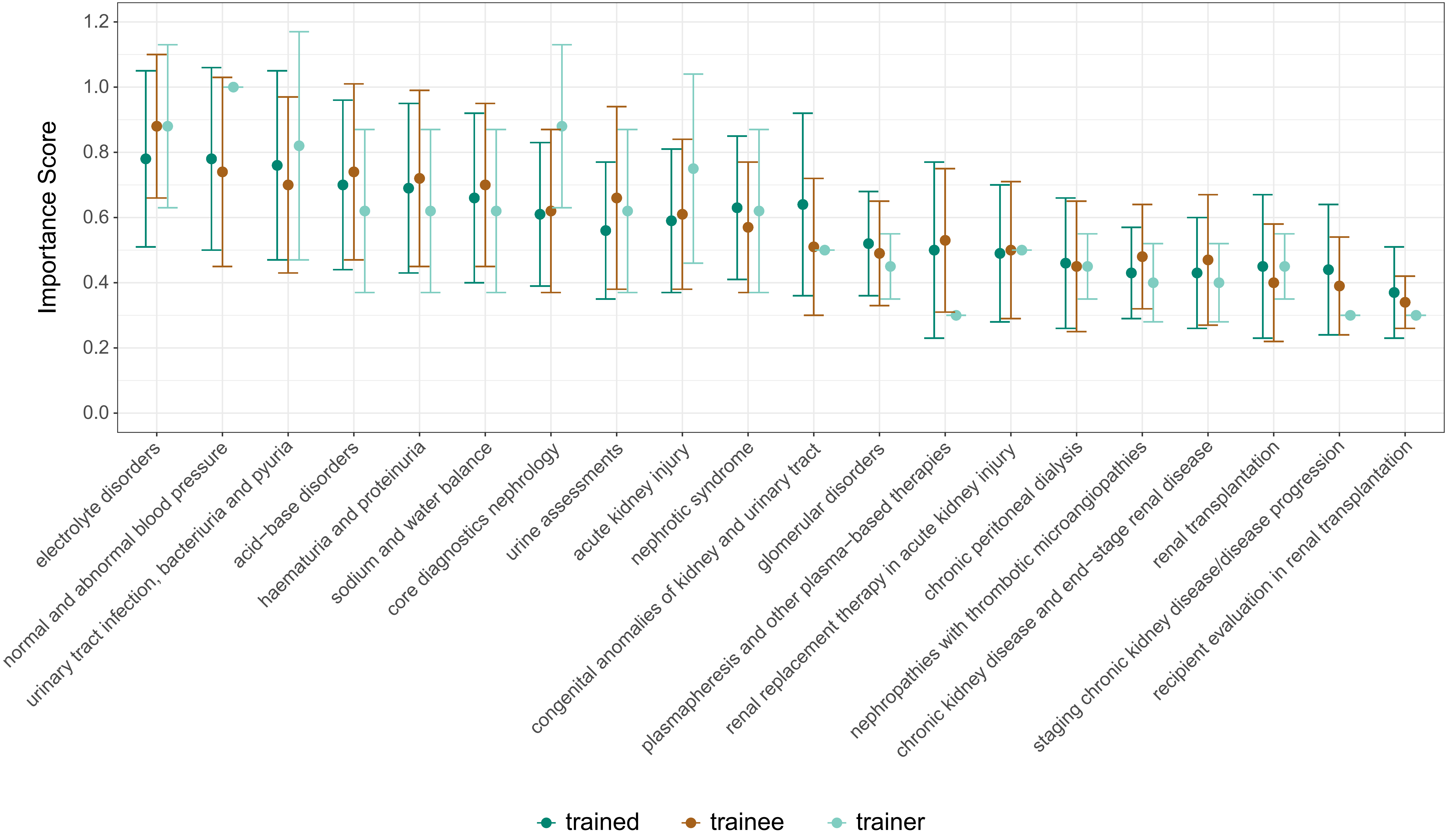

Supplement: Supplementary file 8 [file Image_3.PDF]
