# Supplementary material for: Targeted Training for Subspecialist Care in Children With Medical Complexity
Source: Front Pediatr. 2022 May 16;10:851033. doi: 10.3389/fped.2022.851033 (PMC9149215; doi:10.3389/fped.2022.851033)

Supplemental Figure 2. Importance performance analysis (IPA, = needs assessment) of knowledge themes. “Missing” vs “Learned” dividing panels on the x-axis, mean number of overall occurrences dividing panels on the y-axis. Blue color marks themes of pediatric nephrology.

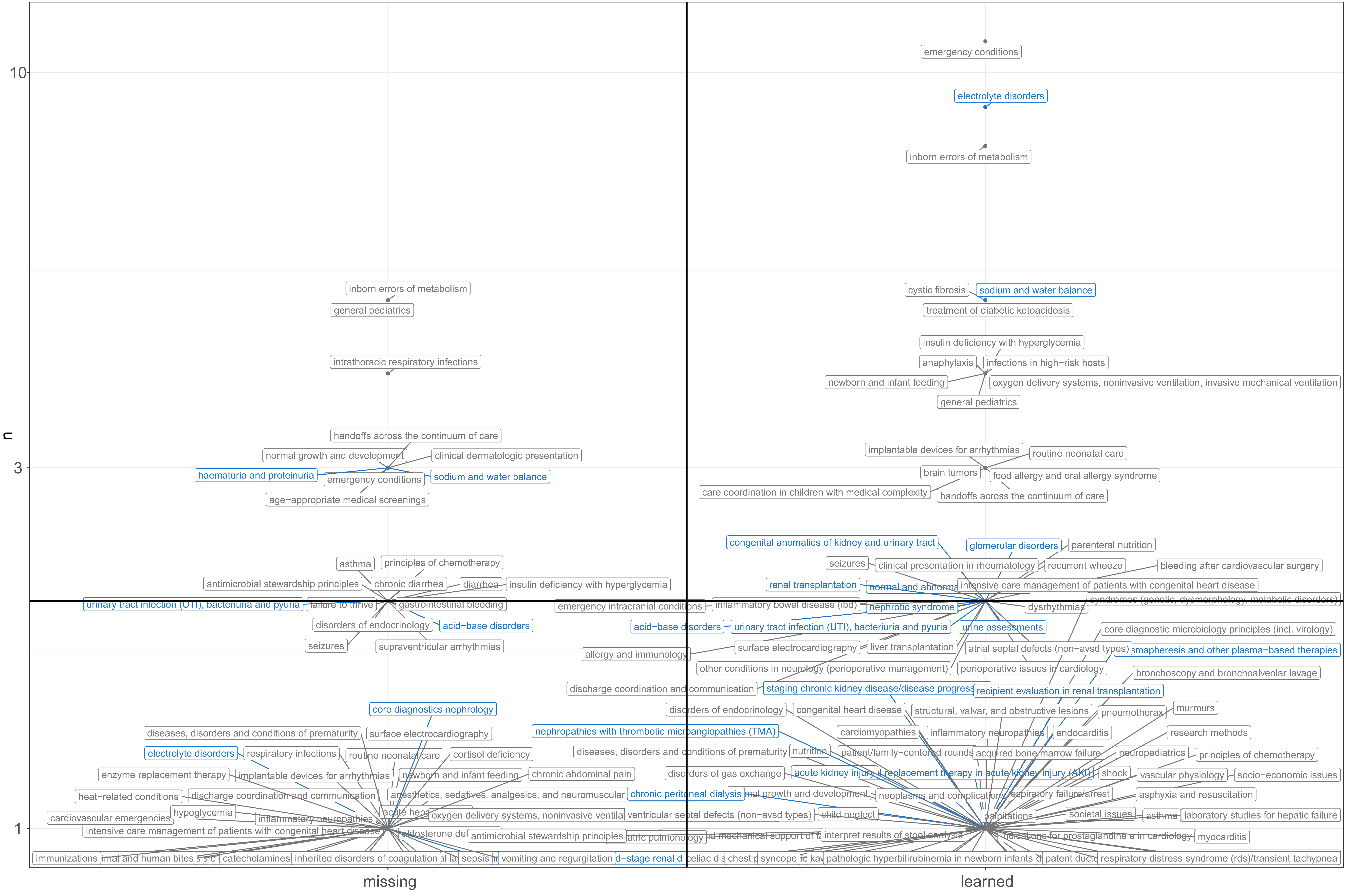

Supplement: Supplementary file 7 [file Image_2.PDF]
